# A philosophy of birth: if you want to change the world, change the conversation

**DOI:** 10.12688/openreseurope.13333.1

**Published:** 2021-06-10

**Authors:** Stella Villarmea

**Affiliations:** 1 Logic and Theoretic Philosophy, Complutense University of Madrid, Madrid, 28040, Spain

**Keywords:** Philosophy of Birth, Standpoint Epistemology, Feminism, Genealogy, Rationality, Capacity, Agency, Logos, Obstetric Violence, Epistemic Injustice, Conceptual Innovation, Gender, Implicit Bias

## Abstract

This essay is about why and how we should introduce birth into the canon of subjects explored by philosophy. Birth care brings to the fore fascinating philosophical questions: is a woman in labour a subject with full rights in practice as well as in theory? Can a labouring woman exercise her autonomy in a situation of maximum vulnerability but also maximum lucidity and awareness, as characterises the work of giving birth? What is the relationship between agency, capacity, and pain during and between contractions? Birth care proposes key questions relating to knowledge, freedom, and what it means to be a human being. Nonetheless, giving birth continues to be a blind spot in contemporary prevailing philosophy.

My approach to a philosophy of birth aligns with one of the aims of contemporary philosophy; I explore the relationship between knowledge and emancipatory action in the relatively unchartered waters of birth and delivery, to create an epistemology that is sensitive to feminism and embodiment. What I propose to achieve through a philosophy of birth is a new *logos* for *genos* —a radically new meditation on origin and birth.

How we understand our origin and the practices that bring us into being reveals our humanity. The lived experiences of women and their *situated knowledge* challenge widely-held assumptions about rationality, about what it is to be a birthing woman and what it is to have agency and capacity in the delivery suite. A philosophy of birth enables us to navigate the stormy waters of contemporary obstetric practice towards a new and radical *logos* for *genos* —an embodied *genealogy* which not only redresses imbalances of gender, but also addresses life and happiness.

## Plain language summary

Every human life begins with gestation and birth. However, in the thought and culture with which I am most familiar —commonly known as Western philosophy— delivery and birth have received considerably less attention than death and mortality. Feminist philosophers and thinkers have criticised this imbalance, rescuing delivery and birth from a state of omission or abandonment. The philosophy of birth constitutes a vibrant and growing field of contemporary critical thought, to which I add my own voice, principally in analysing how much of birth care continues to underestimate the capacity of a woman in labour to behave rationally. Women frequently have their rights breached during childbirth, and we need to know why this happens before we can fix it effectively. This essay takes an innovative approach to explaining how we can protect women´s rights during childbirth.

## Introduction

Every human life begins with gestation and birth. However, in the thought and culture with which I am most familiar —commonly known as Western philosophy—, delivery and birth have received considerably less attention than death and mortality.

Birth is addressed by philosophy both in real terms and as a metaphor in various arguments. Nevertheless, although philosophers have considered questions related to birth, in particular the ethical and metaphysical aspects of the creation of human life, they have paid scant attention to the actual processes of delivery and birth. Even considering the work of Plato ^1^, Arendt ^2^, Schelling ^3^, Freud ^4^, or, most recently, Sloterdijk ^5^, the outcome is the same: we are talking of a deafening silence —a silencing even.

In contrast, mortality has taken central stage in the history of philosophy. There are many thinkers who identify philosophy with learning to die, but relatively few consider birth a subject for philosophy and even fewer give delivery or pregnancy a second thought. In this respect, the Heideggerian expression that characterises human existence —albeit excessively— as ‘being-toward-death’, captures the imbalance that pervades the history of philosophy as we generally know and teach it ^6^.

Feminist philosophers and thinkers have criticised this imbalance, rescuing delivery and birth from a state of omission or abandonment. Their reflections make up a body of varied and opposing thought. I am thinking of Virginia Held ^7^, Adriana Cavarero ^8^, Luisa Muraro ^9^, Sara Ruddick ^10^, Grace Jantzen ^11^, Iris Marion Young ^12^ or Christine Battersby ^13^, from years ago. Or, more recently, of Christina Schües ^14^, Alison Stone ^15^, Elselijn Kingma ^16^, Sara Cohen Shabot ^17^, Sarah LaChance Adams ^18^, Jonna Bornemark ^19^, Andrea O´Reilly ^20^, Luce Irigaray ^21^, Fanny Söderbäck ^22^, Andrea Nye ^23^, Johana Oksala ^24^, Jane Lymer ^25^, Sara Heinämäa ^26^, Lisa Baraitser ^27^ or María Martín ^28^, among others.

The philosophy of birth constitutes a vibrant and growing field of contemporary critical thought, to which I add my own voice.

### Unique experiences

Pregnancy, labour, and birth are unique human experiences, not comparable to others. They involve the creation of a new person by means of a profoundly intimate process that transforms the mind and body of the woman, whether during gestation or in labour, along with her frame of understanding and evaluation of herself and her environment.

The processes of pregnancy, labour, and birth give rise to central philosophical questions in epistemology, metaphysics, political philosophy, and ethics. The field of birth is fertile ground for exploring questions and answers provoked by themes relating to the body, citizenship, health, or technology.

Birth care brings to the fore fascinating philosophical questions: is a woman in labour a subject with full rights in practice as well as in theory? Can a labouring woman exercise her autonomy in a situation of maximum vulnerability but also maximum lucidity and awareness, as characterises the work of giving birth? What is the relationship between agency, capacity and pain during and between contractions? Birth care as a professional field for healthcare personnel and the healthcare system proposes key questions relating to knowledge, freedom, and what it means to be a human being. Nonetheless, giving birth continues to be a blind spot in contemporary prevailing philosophy.

The philosophy of birth can be approached from the perspective of the philosophy of medicine, or from the relatively recent field of the medical humanities. In adopting these perspectives, I see obstetrics as an empirical and symbolic space where contemporary thought reformulates the meanings and metaphors of gestation, labour, delivery and birth. Moreover, a philosophy of birth has additional value when it is based on empirical data and medical practice, and therefore my questions, arguments and philosophical conclusions have as both their starting point and their goal, the current debates within obstetrics. Naturally, approaching birth from a philosophical point of view will reveal new horizons of thought, however, I know from experience that this can also have a real impact on clinical practice. Recently, when she was given a professional award an obstetric colleague expressed her gratitude for what she had learnt from philosophical conversations and mentioned how they had influenced her clinical practice.

Giving birth can be better understood against the backdrop of a complex web of emotions inevitably produced by the relationship between knowledge, power and practice that characterise every situation ^29^. But sometimes we retell events differently to the way in which they happened. In my case, I began using the expression ‘philosophy of birth’ to describe not what I was reading, but what I was thinking; what I wanted and what I needed to think and communicate based on my experiences. It is still important to me to hold on to the memory that identified the genesis of this expression in my life and in my philosophical endeavour.

On the strength of my varied experiences, I became interested in reflecting on what I call ‘the pregnancy of the subject’ or ‘the pregnant subject’ ^30^. I use these formulations to emphasise that the questions which arise when a woman falls pregnant are questions that affect subjects who are subjects in every sense —political, moral, epistemic, sexual or legal—, and who need to be considered from this point forward, as much from a philosophical as from an obstetric perspective.

### A new *logos* for *genos*

I am told that my assertion is radical, but I maintain that our conception of the world and of humans is reflected in the notion of pregnancy, labour, and birth. I am convinced that the rules which govern our view of the world and of human life are compromised by the myriad rules that govern the processes of pregnancy and birth —from medical training to the management of hospitals, from statutory budgets to employment law.

Philosophical reflections on the question of origin have a long history of identifying ‘origin’ with key concepts such as ‘beginning‘, ‘logos’, or ‘foundation’, as developed by the great exponents of the history of philosophy. But what happens when we take the expression ‘rethink the origin’ literally? In philosophy we are not used to associating ‘origin’ — *logos*, *arkhé*, *Ur-*— with ‘birth’, *our* birth.

Rethinking origin means exploring genealogy. I understand ‘genealogy’ in its literal sense, as a *logos* or study of *genos*, in which the Greek term *logos* refers to the word in the sense of meditated, reflected, or reasoned, and the term *genos* refers to the Indo-European root ‘gen-’, which means ‘give birth to’ —as in ‘genesis’ or ‘generate’.

My intention is no less than to rethink the concept of origin itself. What I propose to achieve through a philosophy of birth is a new ‘logos’ for ‘genos’ —a radically new meditation on origin and birth ^31^.

To explore the construction of an alternative genealogy I target obstetrics, one of the paradigmatic places where society constructs discourse around our origin. A philosophical analysis of texts and medical protocols from modernity to the present time reveals the conceptual skeleton of this type of genealogy. What are the hidden assumptions underlying obstetrics and many current practices in pregnancy and birth care? I intend on exposing these assumptions to the light of what philosophers’ call ‘emancipatory conscience’ ^32^.

My approach to the philosophy of birth as genealogy has three distinctive characteristics. Firstly, I differ from many colleagues in this field in that I pay special attention to the moment and the experience of giving birth, rather than to the fact of being born. If you like, I focus on who is giving birth rather than who is being born.

Secondly, in charting the perspective of feminist philosophy to explore these questions in depth, I trace my own nautical map which —like other forms of representation— speaks of a specific context, time, and navigation. Other mappings are possible of course; the waters of the philosophy of birth are open to everyone to sail them as best they can.

Thirdly, my research focuses on a peculiar genealogy —obstetrics; literally a *logos* for *genos*. Understanding how obstetric practice constitutes and expands on contemporary discourse around the construct of origin enables me to formulate a narrative of critical importance around childbirth and birth care practices in health facilities across the world.

Understanding the origin of our discourse on origin is in itself a meta-genealogy. My pursuit of this meta-genealogy aims to identify obstetrics as the science of origin that defines what it is to be human for today. My work on genealogy and meta-genealogy adds to recent philosophical research in the field, in particular to discussions of the genealogy of genealogy by Srinivasan ^33^, Dutilh ^34^, Kail ^35^, Owen ^36^, Haslanger ^37^, and Koopman ^38^; or to the analysis of genealogies as conception of genos by Queloz ^39^, Pettit ^40^, Kitcher ^41^, Fricker ^42^, Williams ^43^, and Craig ^44^. However, while a meta-genealogy is often conceived as a genealogy of the philosophy of genealogy —in other words a genealogy of the views on genealogy by Nietzsche, the Sophists, or any other proponent of the philosophy of genealogy canon— *my* meta-genealogy is conceived as a genealogy of the *science of origin* or rather of obstetrics, as a field of medical science that influences contemporary thought and ways of life in significant ways.

### Patriarchy and obstetric violence

The Spanish philosopher Celia Amorós studies genealogy in the history of philosophy ^45^, and I agree with her claim that philosophical feminism needs to define a genealogy devoid of patriarchal influence ^46^. Personally, I am interested in studying how obstetrics and obstetric practice continues to be significant, as both the source and development of contemporary discourse on the nature of origin. Therefore, I explore a potentially controversial hypothesis: that the very survival of patriarchy itself is closely linked to a certain understanding of and approach to care during pregnancy, labour, and birth. Consequently, I want to extract the philosophical value —both social and political— of the medicalisation of pregnancy, birth, and the post-partum period. If we consider the hypothesis that obstetrics is one of the pillars on which patriarchy solidly rests, then any rational criticism of patriarchy will unavoidably imply a rational criticism both of obstetrics and the assumptions that underpin its daily practice. Analysing the excessive medicalisation of birth is therefore a mandatory step in a contemporary criticism of patriarchal reasoning.

From the point of view of gender, a glance at contemporary science concerned with *genos* shows to what extent obstetrics all too frequently continues to fulfil the function of legitimising the use and abuse of women's bodies, and the mistreatment or violence that many women receive at a critical point in their lives. To understand this, we should be aware of studies and reports by national and international organisations that denounce the tremendously damaging effects of certain unjustified and unjustifiable —but commonplace— practices carried out on women giving birth ^47^.

Violence against women giving birth has become so normalised that it is still not considered as violence against women —almost as if its habitual nature renders the violence invisible. The term ‘obstetric violence’ has been coined to refer to the violence suffered by women in health centres during birth care, and the recent 2019 UN Special Rapporteur's Report on Violence against Women in Reproductive Health Settings ^48^ establishes obstetric violence as a violation of human rights. Obstetric violence, for which we now have data, statistics, and even laws, is one manifestation of gender violence which characterises patriarchy ^49^.

Stories of trauma told by women in testimonies received by the Rapporteur demonstrate how mistreatment and violence against women giving birth in health centres happens *all around the world* and affects women of *any* socio-economic status. Published data confirms that women who are victims of obstetric violence are often silenced or are too frightened to speak out because of taboo, stigma, or the belief that the violence they suffered might just be an isolated incident. However, their testimonies demonstrate that mistreatment and violence during birth are both widespread in practice and deep-rooted in healthcare systems.

Obstetric violence often adopts the form of medical procedures such as episiotomies or caesareans in cases where there is no physiological justification. The practice of carrying out extensive gynaecological examinations during maternity care can cause damage to pregnant women and their babies, and the excessive use of synthetic oxytocin to induce contractions and labour also causes damage to health. Women reported that some health facilities failed to respect their privacy and confidentiality when carrying out vaginal examinations, even performing them in front of third parties. Many women in different parts of the world have described profoundly humiliating practices and verbal aggression that take place behind closed doors in health settings. It is only recently that women have started to talk about the mocking and reproaches, the insults and shouting they have had to put up with from health personnel. Sexist and offensive remarks have been particularly noteworthy. Testimonies of women have described comments such as: ‘You didn't cry when you did it; open your legs or your baby will die, and it will be your fault’ (UN Report 2019: 12).

Women also reported a lack of respect for their autonomy and for their capacity to make decisions, including their choice of birthing position. The UN report tackles the question of informed consent as a human right and as a safeguard against this type of violence. However, frequently women are denied the right to make informed decisions about the care they receive during birth, an omission which constitutes a violation of human rights that their respective states and health systems are responsible and accountable for.

In a nutshell, the report emphasises that all such practices must be identified and treated as violations of women’s human rights.

As an example of the widespread nature of these practices, in 2020 the United Nations convicted Spain of human rights violations in birth care in an historic ruling which serves as a precedent for the rest of the world ^50^. For the first time, a UN legal organisation recognised the existence of obstetric violence and demanded that a state adopt measures to combat it. The ruling confirms obstetric violence as a serious breach of human rights and as an act of discrimination. The discrimination is premised on the fact that since only women can give birth, when women are not granted access to high quality gynaecological and obstetric services they are placed in a position of inferiority and inequality compared to men. The ruling is also ground-breaking in terms of the measures it outlines: it recommends that the Spanish state educates judges and health professionals about obstetric violence and that ‘remedies’ or solutions are put in place so that women should not suffer obstetric violence in Spain. The ruling also recommends that if women do suffer violence, they must be afforded compensation and reparation.

In summary, if we examine the contemporary science concerned with *genos*, we reveal serious questions about power, autonomy, and vulnerability. If maternity services are to become safer, person-centred, and values-based ^51^, we need to view the ethical and legal aspects of women’s birth experiences through the lens of feminism, socio-legal theory, and philosophy. The extent to which women have agency during facility-based childbirth is a critical challenge in our access to full citizenship ^52^.

### Agency and rationality

A philosophy of birth helps to explain why these reports of human rights violations, far from reflecting sporadic incidents or episodes experienced by women during their lives, ‘are part of a continuum of gender-based violence that occurs within a wider context of structural inequality, discrimination, and patriarchy, and is the result of a deficiency of education and training at various levels’ (Villarmea and Kelly 2020: 519) ^53^. As women, we and our children survive detrimental treatment of our bodies and attacks against our decision-making capacity and our freedom of choice, through practices which —most importantly— are carried out without any clinical justification.

Exposing the absurdity of patriarchy in its purest and most basic form as it affects our entry into the world and impacts on those who introduce us to the world is philosophical territory. Philosophy, which Plato called the ‘medicine of the soul’, can use its scalpel to reveal those theories and practices which today still oppress and denigrate women's bodies ^54^.

My work in the philosophical field of birth considers human birth through the lens of the history of the naturalisation of women’s rationality ^55^. I refer to the naturalisation which maintains that biology imposes more restrictions on women's rationality than on men’s, an idea derived from the traditional view of rationality as a human characteristic that is radically distinct from our biological features, let alone from the features that govern reproduction and birth. According to this traditional view, there is, allegedly, an unbridgeable chasm between the spheres of birth and reason.

In essence, the naturalisation of women’s rationality consists in maintaining that women *think* but think less clearly than men because they are more directly affected by irrational biological processes that impair their capacity as rational agents ^56^. The more intense and painful these processes are, the more they impair women’s rational capacity. If the mere presence of a uterus were enough to disturb a woman's reason, it is obvious what will happen when the uterus is working at full capacity during labour, with all the pain that accompanies it. A woman in labour fits the paradigm of irrationality perfectly.

Reconstructing the history of the naturalisation of female rationality is therefore key to evaluating current birth care practices and to identifying the assumptions underlying the reality that in delivery units informed consent is more implied than guaranteed ^57^, and why, despite being enshrined in law, the rational and legal capacity of a woman giving birth is not always upheld in practice.

I will briefly outline an example of the naturalisation of reason in obstetrics; the Enlightenment debate about the ‘thinking uterus’. In the 18th century, medicine, now based on new science and empiricism, was fascinated by the relationship between women's brains and their uteri, and doctors were concerned with understanding how these two organs were connected. An ‘analysis of the “thinking uterus” debate illuminates the different ways in which various arguments were used to justify the subordination of women; sometimes emphasizing the connection between the uterus and thought and sometimes negating it, but always concluding that women’s inferiority is to be found in some known or yet-to-be-discovered anatomical and, primarily sexual, deficiency or problem’ (Villarmea 2021: 22) ^58^.

An unexpected actor intervened in the debate: the famous adventurer Giacomo Casanova, who mocked those arguing over ‘lana caprina’ or nonsense. Casanova maintained that the difference between the reasoning of men and women lies not in their organic make-up, but in their social and educational background. Until now, his staunch defence of the cognitive capabilities of women has received scant attention in specialised studies on the fight for sex and gender equality. My investigation helps to reveal this little-known contribution from Casanova ^59^.

Beyond noting certain prescient moments in the fields of medicine and the history of ideas, I aim to delve deeper into a number of age-old stereotypes that persist in contemporary obstetrics ^60^. Concluding that women are inferior by emphasising the connection between a woman’s uterus and her cognition is an historic argument. The age-old interpretation of the uterus as an organ which transforms women into wild beings, both irrational and uncontrollable, may continue to permeate modern-day obstetrics despite contemporary medicine and science being supposedly free of such prejudices. I believe that a philosophy of birth can be immensely valuable in diagnosing fallacious assumptions and implicit biases ^61^ about the relationship between the womb and reason, and in challenging potentially toxic narratives that have a major impact on birth care.

Even in countries with high standards of living, high quality birth care is inconsistent and frequently falls short in respecting the rights of those it serves. My focus re-evaluates the frontiers of autonomy and legal capacity in the delivery room, using the instruments afforded us by the epistemology of medicine. Through analysing obstetric debates, I describe and prescribe the agency of pregnant women and women in labour using the principles of critical epistemology. Pregnant women and women in labour, a singular and vulnerable group with a basic human right to dignity, also deserve to benefit from the analytical tools of emancipatory philosophy.

I am formulating a philosophy of birth that views birth as an act which is intrinsically governed not only by nature and physiology, but also by cultural interpretations and personal significance. My research tackles the social, legal, and individual context of birth in order to test our notions of agency, autonomy, capacity, rationality, embodiment, and gender. I bring the history of ideas, medical epistemology, and feminist philosophy into close dialogue with our best empirical understanding of life sciences, especially human reproductive physiology, obstetrics, and midwifery. This range of subjects, which I gather under the rubric ‘philosophy of birth’, addresses what I believe is a critical philosophical task.

### Exposing the stereotype

It is well-known that throughout history philosophers have underestimated women's capacity for rationality. They developed ideals and conceptions of reason which were either not designed with women in mind or which directly excluded them. This politically problematic history has both reflected and contributed to systems of injustice ^62^, and we continue to be affected by the practical consequences of these historical narratives. The footprint of these narratives goes far beyond simple disputes within a specific discipline —we still experience the very real impact of these gender stereotypes in childbirth ^63^ and therefore we must address them.

These stereotypes can be observed very clearly in our field of study —the mental capacity of a woman in labour. A woman in labour far from embodies the typical or ideal characteristics of a rational agent. But rationality, which relates to reason, is more than comparing stock market values to decide where to invest your money.

The woman in labour who decides she wants to get up and move around is indeed *rational* since, in order to find an appropriate birthing position, she has evaluated the resources and options available to her. Everyday philosophy calls this practical reasoning or practical use of reason, which involves life experience. I am reminded that nature documentaries view chimpanzees piling up boxes to reach bananas as proof of a cognitive learning process ^64^, while the decisions a woman takes to find a good birthing position are not recognised as a cognitive process. How is it that chimpanzees show a spark of intelligence by climbing on boxes, but a woman moving around during labour is simply ‘following her animal instincts’?

Another example in the case of a woman in labour relates to her cry. A labouring woman’s cry is considered non-rational behaviour; a sign that she has lost control. However, it may be that a woman crying out during labour is being more rational —more *prudent*, in the Aristotelian sense— than we think. Ultimately, perhaps her cry is *premeditated*. After all, a woman in labour is not a being from another world; she is keenly aware that our culture interprets the heart-rending cry of a labouring woman as a paradigm for total loss of control. You only need watch the films. Consequently, in this context, many women think very carefully before releasing loud cries —not to mention they may prefer not to be a nuisance, another typical learned reaction which demonstrates a certain medical socialisation. For these reasons it takes courage to emit the first cry, to try it out, see what happens and if it seems good to continue —‘good’ of course, in relation to what is actually being attempted, i.e. giving birth. But it may simply be that a woman in labour *knows* her cry will help her, because she has *learned* that guttural sounds emitted from one’s throat open the birth canal. There is a direct connection between the muscles of the throat and those of the pelvis —opera singers learn that controlling their pelvic floor helps them reach some of the highest or lowest notes. If this is the case, we will need to start admitting —as hard as it may be— that women may learn, in ante-natal classes or in conversations with friends, that crying out may help them. When the moment arrives, they try it out and it helps. And that is why they continue. They choose, test, evaluate, and confirm —pure method. Why are we so reluctant to acknowledge what they do is *rational*?

Ludwig Wittgenstein explained that the meaning of a sign cannot be innately interpreted; rather, a sign needs the context of social practices to realise its meaning ^65^. A cry uttered during labour need not be any different ^66^. The cry is a sign of something. The patriarchal context interprets it as a lack of control, but the interpretative context may be different: we may interpret the guttural sound as a way of maintaining the rhythm of breathing and working through the pain. Among humans, a cry or guttural sound can have many interpretations; it can be an order, a limit, a lament, a vindication, relief, an impact, a mantra, or an expression of pleasure, to mention a few. Why should it not also be an *intentional* action that opens up an organ to facilitate entry to a unique existential space?

Of course, there are contexts in which the cry means something else, which is why it is so important to remember that the cry is a sign. It can, for example, signify that the birthing woman is expressing her fear or anxiety, her complaint, or protest. Or it might be the way she asks for an epidural. There are even contexts in which the person crying out —the person learning to cry out— is the husband or partner, in a striking and emotional projection of empathy or solidarity: ‘In certain tribes of the Amazon, women still customarily give birth on a river bank. While the woman is labouring, her partner lies in a hammock and cries out, mimicking the pains of child birth. This ritual is intended to trick evil spirits into focusing their attention on the man, thus protecting the woman and child from harm’ (Quintero 2001: 12) ^67^. There are also contexts in which ‘significant others present at childbirth push with the laboring woman, adding themselves to her pushing, supporting her in her efforts without even reflecting on this’ (Cohen Shabot 2020: 10) ^68^. Interestingly, ‘when people are made aware of the fact that they are pushing with the woman in labor, they generally stop pushing, at least for a time’ (La Chance Adams and Burcher 2014: 76) ^69^. Reflecting on the phenomena of ‘communal pushing’ or empathetic sharing of cries during labour would introduce yet another rich perspective in our discourse.

There are other explanations too. Further on during labour, a cry might cease being rational behaviour in the sense of being premeditated to connect a means to an end, or as a learnt resource. With luck, at a certain point during labour, behaviour which started for cultural reasons enters a distinct and interesting phase that I will refer to for simplicity's sake as physiological. Once a woman has tested the virtues of her cry; once she is confident of its value and has used it to transition to the next stage of labour, her scream might become something else, for example a tool for navigation or a —loud— mantra for concentration. Then her cry signals that everything is going well; she feels safe to land on 'planet birth' —a notion that refers to women's descriptions of being or entering into another time zone, space, or even world during labour. ‘As the labour intensified, women withdrew into an inner world where time seemed to be suspended. Women described how this inner space allowed them to concentrate on the labouring process, and this facilitated the feeling that they could manage’ (Olza *et al.* 2020: 6) ^70^. Research shows that ‘women may feel they are worlds apart from people in the same room, that the universe has narrowed to this one task they have to do. Some women mention transcendental experiences; of feeling part of the divine, the universe or gaining a deeper understanding of, or being a part of, nature’ (Olza *et al.* 2018: 4) ^71^. In those contexts, screaming during labour does not mean being ‘ *out* of herself’, but being simply ‘ *in* herself’.

Think of the sound that soldiers make on the battlefield. Initially battle cries may be chants designed to motivate, then shouts to encourage speed, later for focus and finally, the sound of the enemy being targeted. We would not routinely consider soldiers as behaving irrationally when they let out this final cry; we would be more inclined to think of their final sound as fulfilling a function —an appropriate means to achieve a desired end. Let us compare this with how easily in some contexts, the birthing woman’s sounds are taken to mean *just* one thing; that she has lost control and perhaps even her capacity.

Or consider the way in which in some cultures, the pain of grieving for lost loved ones is signified by silent behaviour when in public, while in other cultures mourning is accompanied by or even requires heart-breaking screams and loud wails. Why are we more inclined to think of birthing language as less cultural than, say, mourning language?

As humans, birthing women do the same things as, or similar things to, each other. However, the same —or similar— scream has different meanings depending on the prevailing culture, situation, lifestyle, language game, or worldview where we live, and who, where, when, and for what purpose the scream is uttered **.** We must challenge the univocity of birthing behaviour to allow different and better interpretations *in context*. The scream of the birthing woman is tuned into a specific culture and context and should be interpreted within both. This is what it means to say that the birthing woman´s cry is a sign.

To reduce the multiplicity of meanings and application of labour sounds to a simplistic ‘she does not have capacity’ is a sign of patriarchy. We need to address the multiplicity of voices on childbirth, their autonomy and agency, and we can advance knowledge in this area by using the feminist research frameworks of embodied philosophy to determine what works, for whom and under which circumstances ^72^. In my view, the birth cry is one such voice ^73^.

### Situated epistemology

In the context of a philosophy of birth, I see knowledge as a tool for action. We look for knowledge to indicate or enable courses of action that can move us forward. In approaching a philosophy of birth, I therefore appeal to a pragmatist epistemology. Pragmatists hold that knowing is an ongoing process of inquiry that relies on experience and evidence —we want to have our knowledge confirmed by the force of experience. Those who *know* possess the agency and the capacity to anticipate, imagine and create features in the environment to reach their goals. In my view, knowledge mediates our relationship with the world and is purpose driven. In this way, we can see knowledge as an orientation towards or a response to how to live and act in the world.

In pregnancy and birth, women´s bodies and minds experience a deep transformation. This involves an aspect of suffering in the classical sense of *pathos*, which women experience at many levels and over which they do not necessarily have any choice. But this is also a time of *projection*, *freedom,* and *existence*, in the sense in which these notions exist in the phenomenological or existentialist tradition. The knowledge that is constructed during this time is ‘situated’ knowledge, i.e. knowledge that is concerned with and gives meaning to a time and a place. The theory that reflects on this knowledge must therefore be a ‘situated’ epistemology, which is intrinsically connected to that time and place.

My approach to a situated epistemology of birth aligns with standpoint theory ^74^, a feminist theoretical perspective which argues that knowledge stems from social conditions. In societies stratified by gender, a woman´s social position defines what she can know.

A situated epistemology of birth is therefore material for feminist thought. In my view, being pregnant and giving birth are fertile *standpoints* for knowledge —they allow access to specific knowledge that is not available to those who do not share the experience. Identifying women’s testimonies of obstetric violence as rooted in *situated knowledge* has philosophical significance. This means we recognise the birthing woman has the epistemic *privilege* to transition from a personal to a universal (or general) sphere of interest. It also means she is equipped with the epistemic resources to best interpret her needs, claims, fears, and hopes.

In recent years, Miranda Fricker´s notion of ‘epistemic injustice’ has received considerable philosophical attention ^75^. Epistemic injustice refers to those forms of unfair treatment that relate to issues of knowledge, understanding, and participation in communicative practices. According to Fricker, there are two kinds of epistemic injustice: testimonial injustice and hermeneutical injustice. Testimonial injustice relates to assigning less credibility to the words of some population groups. Hermeneutical injustice is disadvantaging people by misinterpreting their social experiences. Epistemic injustice involves a variety of behaviours and practices, including exclusion and silencing; invisibility and inaudibility; systematic distortion or misrepresentation of people’s meanings or contributions; undervaluing of people’s status or standing in communicative practices; and marginalisation as the result of dysfunctional dynamics, amongst others ^76^.

Women who have suffered obstetric violence also suffer these types of injustice. A situated epistemology of birth will hone the analytical tools to unveil and redress wrongful treatment and unjust structures in meaning-making and knowledge-producing birthing practices ^77^.

### Micro-pill for thought: ‘Unnecaesarean’ as an example of situated epistemology

                                                                                                                                                                              ‘unnecaesarean’

                                                     An unnecessary caesarean, especially one coerced without evidence-based medical grounds.

                                                                                                              E.g: Her doctor convinced her to have an unnecaesarean.

                                                                                                                                               By Unnecaesarean, 17 January 2009

*(Urban Dictionary*, consulted 9 December 2019)

***And the woman said.*** And the woman said: ‘It was not a necessary caesarean —It was an unnecaesarean.’ And the word made sense. And the woman saw that it was good. And the cogs engaged. Humans do not cope well with a lack of explanation, with absurdity or with nonsense; seeking an explanation (wanting to understand, needing to understand, being prepared to understand) is a constituent aspect of human nature. The woman said: ‘unnecaesarean’ and in so doing ‘fermented’ not only the explanation but the action itself as I will explain.

When we talk, we do a number of things. As J. L. Austin argues in his theory of acts of speech, language does not only describe, but also prescribes and even produces ^78^. We describe when we say how things are; we prescribe when we say how things should be; we produce when on making a statement we carry out the action expressed in the statement. This can be more easily understood with the help of familiar examples. If I say: ‘I promise to pay you back the money’, in that moment I am carrying out a promise. The promise did not exist until I made it a reality by expressing it with words. I not only say that I promise but my promise is made a reality, which does not mean that I will fulfil it, only that it exists as a promise from now on. When we register a daughter at the registry office and provide her name, we not only communicate to the official the name that we have chosen for our daughter, but in the act of pronouncing her name when registering her, we *produce* her name socially, i.e. we *name* her. From now on, it will be her name. As these examples show, when we speak we are often doing more than just pronouncing words, we are constructing the furniture (not physical but symbolic and institutional) of the world. That is why John Searle calls this type of linguistic act ‘institutional fact’ ^79^.

Similarly, when I declare: ‘This is an unnecaesarean’, I describe not only a fact —an unnecessary caesarean— but I condemn it and offer it up for public condemnation. To call a surgical intervention ‘unnecaesarean’ is to do three things at the same time: (1) to classify it epistemologically as falling outside evidence-based medical best practice; (2) to condemn it morally and politically as illegitimate; and (3) to show it to be proof of respect owed and not received, condemning it legally.

The way that words are used to clarify their meaning is important. When we say: ‘This was an unnecaesarean’, we attest to the fact and we commit ourselves to explain why we said it. Robert Brandon said that assertive acts —those acts in which we either affirm or negate something— ultimately denote commitment. In affirming that it was an unnecaesarean, I agree to participate in the rational practice of asking why, how, when, where, for whom, by whom, and for what purpose this caesarean is or is not, was or was not, necessary.

In this sense, an essential function of the philosophy of birth is to legitimise (philosophically) certain novel linguistic uses of the three closely interrelated aspects I mentioned earlier: epistemic, ethical, and legal. I will now focus on the philosophical legitimisation of these linguistic practices.

***Imagining, discovering, using, and promoting the use of a term.*** Imagining, discovering, using, and promoting the use of a term is something the speakers of a language do. The idea that it is possible to coin new terms —in this case ‘unnecaesarean’— may give the false impression that anyone can introduce new vocabulary into circulation simply at whim. But language does not work like that. Changes we propose cannot be arbitrary inventions by individuals; they must be based on a rigorous analysis of the way of life and culture of speakers of a language, and proposals must be shoe-horned into existing rules governing expressions.

The reasons for reforming language are practical and pragmatic as well as theoretical. Reform requires political, social, educational, and economic decisions, to name but a few. The success or failure of a women’s human rights project depends on our capacity to clarify the concrete meanings of a good number of relevant terms, based on the way of life of the women involved. Accordingly, specifying that in a particular situation the thing that was called ‘caesarean’ was ‘unnecaesarean’, consists of an unsurpassed linguistic efficiency, which is to say an optimal epistemic, moral, and legal efficiency. A single term thus expeditiously articulates, evaluates, and gives guidance as to the right or wrong of a significant number of events.

The World Health Organisation (WHO) states that the ideal rate for caesarean sections is between 10–15% of all births ^80^. Recent data shows that as many as 27.2% of births in the most developed global regions occur by caesarean section ^81^. We must therefore be concerned that potentially two-thirds of all caesareans carried out in developed regions are not justified on medical grounds. Furthermore, a glance at the data for when most caesareans are carried out reveals that the highest number of interventions take place just before Christmas, Easter, summer, and public holidays ^82^. Given this data, it is reasonable to suggest that the epidemic of caesareans discussed by national and international bodies is in fact, an epidemic of unnecaesareans.

The fact there are so many unnecaesareans as to make the term necessary confronts us with a certain pattern of behaviour within medical practice surrounding delivery and birth. We should be asking for whom and for what purpose does this behaviour exist? Therefore, to talk of unnecaesareans, to name them as such and to *declare* them has extraordinary semantic, pragmatic, and epistemic value. It is true that the semantic value (that which relates to the meaning or sense of the expression) of ‘unnecaesarean’ is identical to the expression ‘unnecessary caesarean’, but the expression ‘unnecaesarean’ has the advantage of communicating the content in a manner at once concise, clear, and to the point. Furthermore, its pragmatic value (the way in which context influences the interpretation of meaning) lies in offering us the following choice: either claim the expression simply denotes bad practice or understand that it points to a level of intervention and control of women's bodies which can only be interpreted as practice that goes beyond mere medical negligence. Finally, the expression also has epistemic value since it discloses both the frequency of hospital malpractice and it highlights when further examples of this practice occur. At this point we might like to pause to consider that if two-thirds of urological operations on male patients were unjustified on medical grounds, would we take it lying down?

***Any language is related to a specific way of life.*** Any language is related to a specific way of life or way of thinking or behaving that is peculiar to a specific community. We cannot change language without at the same time changing the world in which it is embedded. This is the fundamental lesson we learn from emancipatory movements.

It will be some time before we see the expression unnecaesarean included in dictionaries and in common use —possibly as much time as is needed to change the world in which we give birth, and in which our voices are heard ^83^.

In the meantime, activism around birth will continue to use and promote the expression. Yes, that was an unnecaesarean.

### If you want to change the world, change the conversation

As I said at the start of my essay, my focus on the philosophy of birth enquires into the relationship between knowledge and emancipatory action. In my work on normativity and practice in the emancipatory use of language, I apply Wittgenstein’s method from *On Certainty* ^84^ to analyse the conceptual innovation produced by birth rights activism. My thesis is that emancipatory movements do not construct language *ex novo*; rather, they proceed from the understanding of how a particular segment of language works, they eliminate any confusion surrounding certain terms, and they propose a creative usage which illuminates meaning ^85^. This form of intervention certainly gives rise to mental and linguistic good health, but, above all, it contributes to political and moral health.

I welcome Richard Rorty´s interpretation of Wittgenstein which is both hotly debated and also inspiring. Rorty emphasises the function of words as catalysts of change: imaginative uses of language are fundamental to starting to talk in a different way or to imagine a better version of ourselves, as Rorty would say ^86^.

This line of investigation reveals how an emancipatory use of language functions within organisations defending the application of human rights to gestation, birth, and post-partum. In accepting the shared social responsibility that should characterise the evaluation of health care, these birth rights organisations create a space where medical protocol can be debated, where health systems can be reformed and improved, and where a deliberative democracy ^87^ can be built.

Birthing women are autonomous beings and have the right for their autonomy to be protected. To acknowledge the autonomy of mothers is to confirm women’s right to life, health, bodily integrity, and freedom from discrimination, in other words to respect their dignity, equal status, and human rights.

The importance of autonomy, informed consent, and good communication in maternal healthcare decision-making has been confirmed by a landmark court ruling in the United Kingdom, the 2015 Supreme Court decision on *Montgomery v Lanarkshire Health Board* ^88^. The Montgomery ruling led the way in establishing that women have the right to make decisions regarding their care during childbirth. *Montgomery* makes it clear that informed consent is a fundamental principle of health care: anyone receiving medical treatment must agree to undergo that treatment. Particularly significant are Lady Hale’s words: ‘[g]one are the days when it was thought that, on becoming pregnant, a woman lost, not only her capacity, but also her right to act as a genuinely autonomous human being’ ^89^. Words are catalysts of change. *Montgomery* has normalised the pregnant subject as a fully entitled subject.

The philosophy of birth reminds me of an inspirational phrase: if you want to change the world, try to change the conversation ^90^. We are working on it.

## Conclusion

The anthropologist Sheila Kitzinger once said: ‘In any society, the way a woman gives birth and the kind of care given to her and the baby points as sharply as an arrowhead to the key values of the culture’ (Kitzinger 1978: 141) ^91^. In addition, I believe that our very conception of the world and of humankind is reflected in the way we labour and birth.

Reading Olympe de Gouges never fails to inspire me. In the preamble to her *Declaration of the Rights of Woman and of the Citizen* in 1791, de Gouges mirrors the language of the famous 1789 *Declaration of the Rights of Man and of the Citizen* and explains that women, just as men, are guaranteed natural, inalienable, sacred rights, and that political institutions are responsible for protecting these natural rights. She writes:

‘Mothers, daughters, sisters, representatives of the Nation, all demand to be constituted into a national assembly. Given that ignorance, disregard or the disdain of the rights of woman are the only causes of public misfortune and the corruption of governments [they] have decided to make known in a solemn declaration the natural, inalienable and sacred rights of woman; this declaration, constantly in the thoughts of all members of society, will ceaselessly remind them of their rights and responsibilities, allowing the political acts of women, and those of men, to be compared in all respects to the aims of political institutions, which will become increasingly respected, so that the demands of female citizens, henceforth based on simple and incontestable principles, will always seek to maintain the constitution, good morals and the happiness of all’ ^92^.

In publishing her document, de Gouges hoped to expose the failure of the French Revolution to recognise gender equality. However, she was unable to make any lasting impact on the direction of the Revolution. As a result of her writings, de Gouges was accused, tried, and convicted of treason, which resulted in her immediate execution. She was one of only three women beheaded during the Reign of Terror —and the only woman executed for her political writings.

The *Declaration of the Rights of Woman* of 1791 is significant because it focuses attention on a set of feminist concerns or aims, which have yet to be achieved ^93^. In 1989, two centuries after the French Revolution, the philosopher Virginia Held could still claim: ‘Only when the conscious experience of mothers, potential mothers, and mothering persons are taken fully into account can we possibly develop understanding that may someday merit the description *human*’ (Held 1989: 388) ^94^.

How we understand our origin and the practices that bring us into being reveals our humanity. The lived experiences of women and their *situated knowledge* challenge widely-held assumptions about rationality, about what it is to be a ‘birthing woman’ and what it is to have agency and capacity in the delivery suite. A philosophy of birth enables us to navigate the stormy waters of contemporary obstetric practice towards a new and radical *logos* for *genos* —an embodied *genealogy* which not only redresses imbalances of gender, but also addresses life and happiness.

## Further reading

### Further reading one

The literature on the damaging effects of certain unjustified and unjustifiable practices carried out on women giving birth is vast and varied. I have chosen the following studies to illustrate the range of analysis:

Begley K, Daly D, Panda S, Begley C. (2019) Shared Decision Making in Maternity Care: Acknowledging and Overcoming Epistemic Defeaters. *J Eval Clin Pract*: 1–8.

Betrán, Ana Pilar, Marleen Temmerman, Carol Kingdon, Abdu Mohiddin, *et al.* (2018) Interventions to Reduce Unnecessary Caesarean Sections in Healthy Women and Babies. *The Lancet* 392.10155: 1358–68.

De Jonge A, Downe S, Page L, *et al.* (2019) Value based maternal and newborn care requires alignment of adequate resources with high value activities. *BMC Pregnancy Childbirth* 19.428: 428.

London School of Hygiene and Tropical Medicine (2020) *The Lancet Maternal Health Series*. URL: http://www.maternalhealthseries.org/explore-the-series/too-much-too-soon/ (accessed: 8 December 2020).

Morse A., *Maternity Services in England 2013-14* (2014) London: House of Commons Committee of Public Accounts. URL: https://www.parliament.uk/business/committees/committees-a-z/commons-select/public-accounts-committee/news/maternityservices-report/ (accessed 11 November 2019).

NHS England (2016) *National Maternity Review. Better Births. Improving Maternity Outcomes in England*. London: NHS England. URL: https://www.england.nhs.uk/wp-content/uploads/2016/02/nationalmaternity-review-report.pdf (accessed 11 November 2019).

NHS England (2019) *Universal Personalised Care. Implementing the Comprehensive Model*. London, England: NHS. URL: https://www.england.nhs.uk/wpcontent/uploads/2019/01/universal-personalised-care.pdf (accessed 11 November 2019).

Nieuwenhuize MJ, I Korstjens, A de Jonge, R de Vries, A Lagro-Janssen (2014) On Speaking Terms: a Delphi Study on Shared Decisionmaking in Maternity Care. *BMC Pregnancy Childbirth* 14.223: 1–11.

Parliamentary Assembly (2019) Obstetrical and gynaecological violence, *Council of Europe Resolution 2306 (2019)*. URL: http://assembly.coe.int/nw/xml/XRef/Xref-XML2HTML-EN.asp?fileid=28236&lang=en (accessed 8 December 2020).

Recio, A., and J. M. Arranz (2020) Evaluación del Impacto de la Estrategia de Atención al Parto Normal sobre las Tasas de Cesáreas y Mortalidad Perinatal en España [An Impact Evaluation of the Strategy for Normal Birth Care on Cesarean Sections Rates and Perinatal Mortality in Spain]. *Papeles de Trabajo del Instituto de Estudios Fiscales*, Serie economía 8: 1–38.

Sokol, D (2014) Let's Stop Consenting Patients. *BMJ* 348: g2192.

The Royal College of Obstetricians and Gynaecologists (2017) *Obstetrics and Gynaecology Workforce Report 2017*. London: The Royal College of Obstetricians and Gynaecologists.

World Health Organisation (2018) WHO Recommendations: Intrapartum Care for a Positive Childbirth Experience. Geneva: WHO.

### Further reading two

Again, literature on obstetric violence is vast so I offer here a selection of references to reflect the breadth of approaches:

Baker, SR *et al.* (2005) ‘I Felt as Though I’d Been in Jail’: Women’s Experiences of Maternity Care during Labour, Delivery and the Immediate Postpartum. *Feminism & Psychology* 15(3): 315–342.

Beck, Cheryl Tatano (2004) Post-Traumatic Stress Disorder Due to Childbirth: The Aftermath. *Nursing Research*, 53.4: 216–224.

Bellón, Silvia (2015) La Violencia Obstétrica desde los Aportes de la Crítica Feminista y la Biopolítica. *Dilemata* 18: 93–111.

Bohren, Meghan *et al.* (2015) The Mistreatment of Women during Childbirth in Health Facilities Globally: a Mixed-Methods Systematic Review. *PloS Medicine*, 12.6: e1001847.

Charles, Sonya (2013) Disempowered Women? The Midwifery Model and Medical Intervention. In *Coming to Life: Philosophies of Pregnancy, Childbirth and Mothering*, ed. Sarah LaChance Adams and Caroline Lundquist, 215–240. New York: Fordham University Press.

Cohen Shabot, Sara (2020a) We Birth with Others: Towards a Beauvoirian Understanding of Obstetric Violence. *The European Journal of Women´s Studies* 24.2: 128–142; (2020b) Why ‘Normal’ Feels So Bad: Violence and Vaginal Examinations during Labour – A (Feminist) Phenomenology. *Feminist Theory*, DOI: https//doi.org/10.1177/1464700120920764; Cohen Shabot, Sara and K Korem (2018) Domesticating Bodies Through Shame: Understanding the Role of Shame in Obstetric Violence. *Hypatia* 33.3: 384–401; and Cohen Shabot, Sara (2016) Making Loud bodies ‘Feminine’: A Feminist-Phenomenological Analysis of Obstetric Violence. *Human Studies* 39.2: 231–247.

Elmir, Rakime *et al.* (2010) Women’s Perceptions and Experiences of a Traumatic Birth: a Metaethnography. *Journal of Advanced Nursing*, 66.10: 21422153.

Fernández Guillén, Francisca (2015) ¿Qué es la Violencia Obstétrica? *Dilemata* 18: 113–128.

Murray, Claire (2020) Troubling Consent: Pain and Pressure in Labour and Childbirth. In *Women’s Birthing Bodies and the Law: Unauthorised Medical Examinations, Power and Vulnerability*, ed. Camilla Pickles and Jonathan Herring, 155–170. Oxford: Hart.

Pickles, Camilla (2020) When ‘Battery’ is not Enough. In *Women’s Birthing Bodies and the Law: Unauthorised Medical Examinations, Power and Vulnerability*, ed. Camilla Pickles and Jonathan Herring, 127–142. Oxford: Hart; and (2019) Leaving Women Behind: The Application of Evidence-based Guidelines, Law, and Obstetric Violence by Omission. In *Childbirth, Vulnerability and Law: Exploring Issues of Violence and Control*, ed. Jonathan Herring and Camilla Pickles, 140–160. New York: Routledge.

Recio, Adela (2015) La Atención al Parto en España: Cifras para Reflexionar sobre un Problema [Birth Care in Spain: Figures for Reflection]. *Dilemata* 18: 13–26.

Sadler, Michelle *et al.* (2016) Moving beyond Disrespect and Abuse: Addressing the Structural Dimensions of Obstetric Violence. *Reproductive Health Matters*, 24.47: 47–55.

Thomson, Gill and Soo Downe (2008) Widening the Trauma Discourse: The Link between Childbirth and Experiences of Abuse. *Journal of Psychosomatic Obstetrics and Gynecology*, 29.4: 268–273.

WHO (2006) *Pregnancy, Childbirth, Postpartum and Newborn Care: A Guide for Essential Practice*. Geneva: World Health Organization.

## Data availability statement

No data are associated with this article.
