# Supplementary material for: A philosophy of birth: if you want to change the world, change the conversation
Source: Open Res Eur. 2021 Jun 10;1:65. [Version 1] doi: 10.12688/openreseurope.13333.1 (PMC10445893; doi:10.12688/openreseurope.13333.1)
Supplement: Peer review history

# Peer review: Reviewer response for version 1

**DOI:** 10.21956/openreseurope.14403.r28129

**Published:** 2022-01-12

**Authors:** Tanja Staehler

**Affiliations:** 1 School of Media, Arts and Humanities, University of Sussex, Brighton, UK

This is a sincere, engaged and thoughtful discussion of a topic which has been under-researched for a long time and is now luckily receiving more attention. The author provides a very helpful, comprehensive bibliography which can also be useful for future research in this area. The argument is persuasive and well structured. It is particularly unusual and impressive to see resources from analytic philosophy utilised for an investigation of childbirth.

 There are a few formulations which I would like the author to clarify if they agree:

1. "I am told that my assertion is radical, but I maintain that our conception of the world and of humans is reflected in the notion of pregnancy, labour, and birth.” – To my mind, these should be two sentences, and I would like to hear more clearly which assertion is radical.
2. **“**Thirdly, my research focuses on a peculiar genealogy —obstetrics; literally a *logos* for *genos*.” This makes it sound as if obstetrics literally meant ‘logos for genos’, which is obviously not the case. It would be good to reformulate. The etymology of obstetrics is also interesting, by the way.
3. When it comes to the idea and concept of genealogy, I recommend Alison Stone’s article “Essentialism and Anti-Essentialism in Feminist Philosophy” (DOI: https://doi.org/10.1177/174046810400100202).
4. Undertaking this kind of work with the help of M. Fricker, L. Wittgenstein, etc. is original and convincing. If possible, I would have liked Fricker’s concepts regarding epistemic injustice to be applied even more fully.

Is the argument persuasive and supported by appropriate evidence?

Yes

Is the work clearly and cogently presented?

Yes

Is the topic of the essay discussed accurately in the context of the current literature?

Yes

Does the essay contribute to the cultural, historical, social understanding of the field?

Yes

Reviewer Expertise:

NA

I confirm that I have read this submission and believe that I have an appropriate level of expertise to confirm that it is of an acceptable scientific standard.

## References

[1] : Essentialism and Anti-Essentialism in Feminist Philosophy. *Journal of Moral Philosophy* .2004;1(2) : 10.1177/174046810400100202 135-153 10.1177/174046810400100202

# Peer review: Reviewer response for version 1

**DOI:** 10.21956/openreseurope.14403.r28131

**Published:** 2021-12-22

**Authors:** Sonya Marie Charles

**Affiliations:** 1 Philosophy and Comparative Religion, College of Liberal Arts and Social Sciences, Cleveland State University, Cleveland, OH, USA

Overall, this is a very interesting and engaging article that adds some new and interesting arguments to the discussion. However, there are a few parts that could be more clear.

 First, it seems to me that the main point of this essay is epistemological. Western philosophy has denigrated women's rationality, birth has played a key role in how that has been articulated in Western philosophy, and now we see it translated in to things like obstetrical violence. Admittedly, this is an overgeneralization of some of the more interesting arguments the author makes.  However, I feel this summary captures the thread that runs through the main arguments. I would make this point a bit sharper in the abstract. Some of the arguments about how this research project "reveals our humanity" is less clear to me as I work through the paper. For example, when the author discusses the three distinctive characteristics of their philosophy of birth as genealogy, they say "I pay special attention to the moment and the experience of giving birth, rather than to the fact of being born." For me, this shows that the main focus is the epistemological question and how that relates to woman's social and political status and less about our shared humanity.  

 Second and related, the two sections that could use a bit more clarity were: "A new logos for genos" and "agency and rationality." I will admit that I am not well-versed in the use of genealogy as a philosophical tool. I have read some authors who use it, but I do not have a strong meta-understanding of it. Therefore, my confusion in this section, could be my own. Still, while I understood the general flow of this section, it was unclear to me how this related to the rest of the argument.

 Still, the section that seems to need the most clarification is  "agency and rationality."  What the author says here seems correct, but it is very vague. Perhaps, more importantly, the author introduces ideas without sufficient follow-up. For example, the author says, "I will briefly outline an example of the naturalisation of reason in obstetrics; the Enlightenment debate about the 'thinking uterus.'" The paragraph goes on to say some general things about this topic. I took this as an introduction to the topic not the entire discussion. Yet, the next paragraph pivots to a contradiction of this view and the paragraph after moves on to a new subject. At the very least, this discussion moves too quickly and needs more support and/or transitions.

 Aside from these specific comments, I found the rest of the paper very engaging. From "exposing the stereotype" on, the author presents a very useful epistemological analysis. The diverse ways the author approaches this question/issue was insightful.  It definitely illustrates the author's goal of showing how studying birth can be a useful philosophical project--especially to areas such as philosophy of language (which might consider this topic irrelevant).

 In sum, I think this is a useful and well-argued paper. My early comments are only meant to be as precise as possible in pointing out areas for clarification. Overall, I believe the author's argument is efficacious and adds to the on-going literature.

Is the argument persuasive and supported by appropriate evidence?

Yes

Is the work clearly and cogently presented?

Partly

Is the topic of the essay discussed accurately in the context of the current literature?

Yes

Does the essay contribute to the cultural, historical, social understanding of the field?

Yes

Reviewer Expertise:

bioethics (especially reproductive ethics), ethics, social and political philosophy

I confirm that I have read this submission and believe that I have an appropriate level of expertise to confirm that it is of an acceptable scientific standard, however I have significant reservations, as outlined above.

# Peer review: Reviewer response for version 1

**DOI:** 10.21956/openreseurope.14403.r27077

**Published:** 2021-08-26

**Authors:** Sarah LaChance Adams

**Affiliations:** 1 Department of Philosophy and Religious Studies, University of North Florida, Jacksonville, FL, USA

Response to Stella Villarmea’s “A Philosophy of Birth: If you want to change the world change the conversation”

 “A Radically New Meditation for Philosophy”

 Sarah LaChance Adams

 University of North Florida

 As Stella Villarmea notes, philosophy has often taken death as its existential foundation. This is due in no small part to the exclusion of women from the practice of philosophy.[1] It would indeed be a radical shift to reorient its conversation toward birth, specifically toward the origin of life in the female body.[2] Villarmea rightfully “pay[s] special attention to the moment and experience of giving birth, rather than to the fact of being born. If you like, [she] focus[es] on who is giving birth rather than who is being born.”[3] Even classical psychoanalysts, who many of us include among the philosophers, consider the mother-child relationship only from the perspective of the (adult) child, and for the purpose of understanding the mother’s role in causing psychopathology.[4] In bold contrast to this history, Villarmea insists that the birthing woman is not only the author of her own experience, but also a philosophical authority.

 Some philosophers will dispute that Villarmea’s focus on birth can fundamentally challenge epistemology or even obstetrics. Perhaps they will grant that it may bring new knowledge, but only data that can be added to what they already know. This would be to misunderstand the origins of these disciplines. While this is not the place for a detailed account, it has been thoroughly argued elsewhere that the presumption of women’s irrationality is intertwined with the origin of obstetrics.[5] For instance, scholars have demonstrated that many women accused of witchcraft were, in fact, midwives. Since their principles and methods were not understood via religious or scientific methods, they were deemed magical or supernatural. The scientific method was likened to a witch trial and “the secrets of nature [were] imagined as deliberately and slyly ‘concealed’ from the scientist.”[6] Kramer and Sprenger, authors of the notorious *Malleus Maleficarum *(1486), said that midwives were the worst of the witches. Progressively, midwifery was overtaken by obstetrics, described as “the gradual attempt by man to extricate the processes of birth from women and call it his own.”[7] Francis Bacon’s decree to rule over disorderly nature with masculine force was underway. “What has been especially brought to light is what now appears as a virtual obsession with the untamed natural power of female generativity, and a dedication to bringing it under control.”[8] Given this history, to take the birthing woman’s guttural cry as rational and authoritative poses a radical challenge to the foundations of scientific inquiry.

 Doubt and anxiety have been part of philosophy’s epistemological disposition at least since Descartes’ *Meditations *(1637). Descartes worries: What if I am hallucinating? What if I am dreaming? What if god is really an evil demon? His misgivings about the shaky ground for his knowledge were justified, but not for the reasons he thought. Modern philosophy’s first principles were to pursue knowledge as an isolated individual, ignoring the social and political situation, and all relations of power and economics. In truth, no one can, or does, do this. In contrast, feminist epistemologists have demonstrated how fundamentally our context impacts our “objectivity” and the further practices that follow from them. For example, Alcoff argues that we should examine our pretenses to neutrality and observe the “fact that scientific knowledge emerges from social praxis that occurs in the interface between scientific and political/economic institutions, and that the latter have determinate effects not only on the principles of research but on the hypotheses that are considered plausible.”[9] Sound knowledge requires an inclusive dialogue that is cognizant of the power dynamics that may be influencing its conclusions.

 The conversation that Villarmea calls for—medical humanities—advances this critical inquiry, which is badly needed to combat the notion of pregnant bodies as passive containers for fetuses. Consider Emily Martin’s devastating and humorous critique of gender biased accounts of human reproduction. They describe sperm as heroes that carry out a “perilous journey” into the “warm darkness,” where some fall away “exhausted.” “Survivors” “assault” the egg and the successful candidates surround the prize. The egg is “a dormant bride awaiting her mate’s magic kiss, which instills the spirit that brings her to life.”[10] I am reminded of a joke in the Monty Python’s movie *The Meaning of Life*. A woman is going into labor among several male obstetricians who are more interested in “the machine that goes ping” than in the woman herself. When she asks them “what do I do?” One doctors replies, “nothing dear, you’re not qualified.”

 Villarmea highlights how the presumed passivity and irrationality of women leads to widespread obstetric violence—a variety of invasive, humiliating, and terrifying medical procedures.[11] Villarmea’s question “Is a woman in labour a subject with full rights in practice as well as in theory?” *ought* to be superfluous; but it is a very practical question indeed.[12] Scholars provide ample evidence that medical interventions are undertaken against women’s consent and knowledge. For instance, Villarmea estimates that as many as two-thirds of caesarean sections are not medically necessary, a claim that is bolstered by the observation that the highest number take place before holidays and summer vacation.[13] Although the World Health Organization and the United Nations have begun to acknowledge the existence of obstetric violence as a human rights issue, it remains largely invisible.

 Clearly, where human rights are violated, there is a dire problem. They dictate our formal obligations to each other and assert our fundamental equality and dignity as human beings. Nevertheless, I think, and I believe that Villarmea would agree, birthing women deserve further consideration. The ethical and political tradition that provides theoretical foundations for human rights are based on a notion of human beings that “sprung out of the earth, suddenly, like mushrooms, come to full maturity, without all kinds of engagement with each other.” (Hobbes, 1966, p. 109, as quoted in Benhabib, 2008, p. 483). They are not born and are never dependent on anyone else. This tradition lacks Villarmea’s *logos* for *genos.*[14]

 Seyla Benhabib offers an alternate paradigm to the prevailing Western tradition, an *ethos* that is more consistent with Villarmea’s genealogy. Benhabib claims that human rights consider us as *generalized* others, a way to equality that can overshadow important distinctions; while she advocates that we treat one another as *concrete* others.

 The standpoint of the concrete other…requires us to view each any every rational being as an individual with a concrete history, identity, and affective-emotional constitution. Our relation to the other is governed by norms of equity and complementary reciprocity: each is entitled to expect and to assume from the other forms of behavior through which the other feels recognized and confirmed as a concrete individual being with specific needs, talents, and capacities…The moral categories that accompany such interactions are those of responsibility, bonding, and sharing.[15]

 Benhabib provides a complimentary *ethos* to Villarmea’s genealogy. The perspective of the concrete other recognizes individuality, perspective-sharing, cultural diversity, and relational-subjectivity. It is at work in friendship, solidarity, and caring. Benhabib can help us to understand the alienation of pregnant women who are treated in a standardized manner, as “rushed” and “processed” through medical appointments, not even knowing if their own obstetrician will be attending their birth.[16] Moreover, it can make greater sense out of problems such as disparities in maternal outcomes within countries such as the United States where Black, American Indian, and Alaska Native women experience up to 3.2 times higher rates of pregnancy-related mortality than white women.[17] Given that they are attended by obstetrician–gynecologists certified by the American College of Obstetricians and Gynecologists (ACOG) we might presume that their outcomes would be more comparable. ACOG largely attributes these disparities to systemic economic inequalities—a *generalizable* problem beyond their control. However, they do acknowledge that practitioner’s social and demographic biases may impact their recommendations for cesarean delivery. In addition, they explain that cultural and language differences may  cause “subtle ambiguities in practitioners’ and patients’ interpretations of medical information” and that a “culturally derived mistrust of the health care system may result from legacies such as coercive sterilization of poor women of color.” It is hopeful to see ACOG recognize the connection between improved health outcomes and attention to the *concrete* differences between patients, even if the specific solutions that they offer are strikingly unimaginative.[18]

 In this article, Villarmea offers a provocative hypothesis: “that the very survival of patriarchy itself is closely linked to a certain understanding of and approach to care during pregnancy, labour, and birth”[19] I want to follow her boldness and propose that one of the ways patriarchy prospers is to divide women (and other genders) by class, race, geography, nationality, ability, culture, and more. When it comes to obstetric violence, many women are rightly concerned with overmedicalization such as *unnecaesareans* and forced sterilization. Meanwhile other women receive little to no medical care at all, such as those in Sub-Saharan Africa and Southern Asia, where approximately 86% of maternal deaths in the world occur.[20] Feminist resistance requires continually uncovering our ignorance about the injustices faced by women of other social locations. As Villarmea writes: “We need to address the multiplicity of voices on childbirth, their autonomy and agency, and we can advance knowledge in this area by using the feminist research frameworks of embodied philosophy to determine what works, for whom and under which circumstances.”[21]

 Works Cited

 Alcoff, L. M. (2008). How is Epistemology Political? In A. B. Cuomo, *The Feminist Philosophy Reader* (pp. 705-718). Boston: McGraw Hill.

 Bacon, F. (2000). *The New Organon.* New York: Cambridge University Press.

 Benhabib, S. (2008). The Generalize and the Concrete Other. In A. B. Cuomo, *The Feminist Philosophy Reader* (pp. 478-496). New York: McGraw Hill.

 Bordo, S. (1987). *The Flight to Objectivity: Essays on Cartesianism and Culture.* New York: SUNY Press.

 Easlea, B. (1980). *Witch-Hunting, Magic and the New Philosophy.* Atlantic Highlands, N.J.: Humanities Press.

 English, B. E. (1979). *For Her Own Good.* New York: Anchor/Doubleday.

 Hobbes, T. (1966). Philosophical Rudiments COncerning Government and Society Vol. II. In E. S. Molesworth, *The English Works of Thomas Hobbes.* Darmstadt.

 Johnson, C. (2013). The Political "Nature" of Pregnancy and Childbirth. In S. L. Lundquist, *Coming to LIfe: Philosophies of Pregnancy, Childbirth and Mothering *(pp. 193-214). New York: Fordham.

 LaChance Adams, S. (2014). *Mad Mothers, Bad Mothers, and What a 'Good' Mother Would Do: The Ethics of Ambivalence.* New York: Columbia University Press.

 Rich, A. (1976). *Of Woman Born.* New York: Bantam.

 Villarmea, S. (2021). A philosophy of birth: if you want to change the world, change the conversation. *Open Research Europe*.

 Villarmea, S. (2021). Reasoning from the Uterus: Casanova, Women's Agency, and Philosophy of Birth. *Hypatia: A Journal of Feminist Philosophy, 36*(1), 22-41.

 Wolf, N. (2001). *Misconceptions: Truth, Lies, and the Unexpected on the Journey to Motherhood.* New York: Anchor.

 [1] “’Philosophy is for posh, white boys with trust funds’ Why are there so few women in philosophy?” Rebecca Ratcliffe and Claire Shaw, The Guardian, January 5, 2015, https://www.theguardian.com/higher-education-network/2015/jan/05/philosophy-is-for-posh-white-boys-with-trust-funds-why-are-there-so-few-women Accessed August 11, 2021

 [2] By referencing the female body here, I am strictly referring to the reproductive organs that are typically considered female in human and non-human animals—uterus, eggs, and so on. I am not asserting that gestational bodies must be “female” in any other respect. Nevertheless, some of the issues here may arise from the identification of pregnant bodies as female and what it means, culturally, to be identified as a woman or girl. These issues are obviously far more complex that I can discuss here, but I hope to undertake them in future work.

 [3] (Villarmea, A philosophy of birth: if you want to change the world, change the conversation, 2021, p. 5)

 [4] (LaChance Adams, 2014, p. 34)

 [5] See for example: Villarmea, Reasoning from the Uterus: Casanova, Women's Agency, and Philosophy of Birth, 2021. See also note 4.

 [6] (Rich, 1976, p. 121) (English, 1979) (Bordo, 1987, p. 128) (Easlea, 1980, p. 214 as quoted in Bordo, 1987, p. 109)

 [7] (Arms, quoted in Rich, 1976, p. 90)

 [8] (Bordo, 1987, p. 109)

 [9] (Alcoff, 2008, p. 706)

 [10] Emily Martin, “The Egg and the sperm: How science has constructed a romance based on

 stereotypical male-female roles,” *Signs* Vol. 16 No. 3 (1991): 485-501.

 [11] (Villarmea, A philosophy of birth: if you want to change the world, change the conversation, 2021, p. 6)

 [12] (Villarmea, A philosophy of birth: if you want to change the world, change the conversation, 2021, p. 1)

 [13] (Villarmea, A philosophy of birth: if you want to change the world, change the conversation, 2021, p. 12)

 [14] (Villarmea, A philosophy of birth: if you want to change the world, change the conversation, 2021, p. 4)

 [15] (Benhabib, 2008, pp. 485-6)

 [16] (Johnson, 2013, p. 212)

 [17] https://www.cdc.gov/media/releases/2019/p0905-racial-ethnic-disparities-pregnancy-deaths.html

 Accessed August 17, 2021

 [18] https://www.acog.org/clinical/clinical-guidance/committee-opinion/articles/2015/12/racial-and-ethnic-disparities-in-obstetrics-and-gynecology

 Accessed August 24, 2021

 [19] (Villarmea, A philosophy of birth: if you want to change the world, change the conversation, 2021, p. 5)

 [20] https://www.who.int/news/item/19-09-2019-maternal-deaths-decline-slowly-with-vast-inequalities-worldwide

 Accessed August 17, 2021

https://www.who.int/news/item/16-08-2016-true-magnitude-of-stillbirths-and-maternal-and-neonatal-deaths-underreported

 Accessed August 20, 2021

 [21] (Villarmea, A philosophy of birth: if you want to change the world, change the conversation, 2021, p. 10)

Is the argument persuasive and supported by appropriate evidence?

Yes

Is the work clearly and cogently presented?

Yes

Is the topic of the essay discussed accurately in the context of the current literature?

Yes

Does the essay contribute to the cultural, historical, social understanding of the field?

Yes

Reviewer Expertise:

Feminist Philosophy, Philosophy of Motherhood, Ethics, Existential Phenomenology

I confirm that I have read this submission and believe that I have an appropriate level of expertise to confirm that it is of an acceptable scientific standard.
